# Comparing study features is easy but identifying next steps is hard: Evaluating critical thinking through the Biology Lab Inventory of Critical Thinking in Ecology

**DOI:** 10.1002/ece3.10071

**Published:** 2023-05-10

**Authors:** Ashley B. Heim, David Esparza, Natasha G. Holmes, Michelle K. Smith

**Affiliations:** ^1^ Department of Ecology and Evolutionary Biology Cornell University Ithaca New York USA; ^2^ Laboratory of Atomic and Solid State Physics Cornell University Ithaca New York USA

**Keywords:** assessment, biology, critical thinking, ecology, next steps, research group comparison, undergraduate teaching, validation

## Abstract

Critical thinking, which can be defined as the evidence‐based ways in which people decide what to trust and what to do, is an important competency included in many undergraduate science, technology, engineering, and mathematics (STEM) courses. To help instructors effectively measure critical thinking, we developed the Biology Lab Inventory of Critical Thinking in Ecology (Eco‐BLIC), a freely available, closed‐response assessment of undergraduate students' critical thinking in ecology. The Eco‐BLIC includes ecology‐based experimental scenarios followed by questions that measure how students decide on what to trust and what to do next. Here, we present the development of the Eco‐BLIC using tests of validity and reliability. Using student responses to questions and think‐aloud interviews, we demonstrate the effectiveness of the Eco‐BLIC at measuring students' critical thinking skills. We find that while students generally think like experts while evaluating what to trust, students' responses are less expert‐like when deciding on what to do next.

## Graphical abstract

To help instructors effectively measure critical thinking, we developed the Biology Lab Inventory of Critical Thinking in Ecology (Eco‐BLIC), a freely available, closed‐response assessment of undergraduate students' critical thinking in ecology. Using student responses to questions and think‐aloud interviews, we demonstrate the effectiveness of the Eco‐BLIC at measuring students' critical thinking skills. We find that while students generally think like experts while evaluating what to trust, students' responses are less expert‐like when deciding on what to do next.

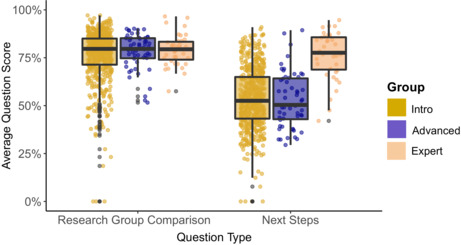

## 1 INTRODUCTION

Critical thinking is an important learning goal of postsecondary education (Bissell & Lemons, 2006; Yuretich, 2004). Students need critical thinking skills—defined here as the process by which people use “data and evidence to make decisions about what to trust and what to do” (p. 1, Walsh et al., 2019)—in both academic and nonacademic settings to effectively make decisions to acquire and evaluate data (Stein et al., 2007). Employers dependably rank critical thinking as one of the most important and necessary outcomes of undergraduate degree programs, as these skills are associated with employees who can consistently make evidence‐based decisions in their careers (Gencer & Dogan, 2020; Murawski, 2014).

Ecology courses provide important settings in which to study and develop undergraduates' critical thinking skills. Notably, the Ecological Society of America's Four‐Dimensional Ecological Education framework includes designing and critiquing investigations as essential practices for ecology courses (Berkowitz et al., 2018; Moore, 1993). In terms of *what to trust*, students explore how scientific data can be messy, with low *R*‐squared values, small sample sizes, or data types prone to systematic issues that can make drawing conclusions difficult (Karban et al., 2014; Kjelvik & Schultheis, 2019). Because current issues in ecology can often impact public discourse (e.g., climate change and biodiversity), it is important that students learn how to evaluate the trustworthiness of data (McCright, 2011). Students learn about *what to do* by controlling confounding variables, making inferences, and distinguishing between correlation and causation (Bonner et al., 2017; Kjelvik & Schultheis, 2019; Mourad et al., 2012). Students also learn that experimental manipulations can sometimes be impossible due to ethical or logistical constraints (Karban et al., 2014).

### 1.1 Assessment framework

One way to help instructors measure critical thinking in ecology courses is to provide evidence‐based assessment instruments that focus on critical thinking. To date, most studies that assessed critical thinking in ecology field courses used student self‐reports (e.g., writing reflections and self‐assessment of their learning gains; McLaughlin et al., 2018) or qualitative evidence of critical thinking gains (Gillie & Bizub, 2012). Instructors could use several STEM (i.e., science, technology, engineering, and mathematics) assessments to measure critical thinking in their courses (Table 1). These instruments include content ranging from broad STEM to biology‐specific topics. Many of the assessments, however, are open‐response and may be challenging to score with large classes. In addition, a key design choice in critical thinking assessment is the inclusion of questions that explicitly probe students' evaluations of what to trust and what to do, which aligns with the definition of critical thinking from Walsh et al. (2019). Several assessments probe student understanding of data and methods (“What to trust”), but few also ask students to evaluate proposed next steps in a scientific investigation (“What to do”; Table 1).

**TABLE 1:** Design principles of existing critical thinking and experimental design assessments that could be used in biology courses.

	Skills/concepts assessed		Structure			
Assessment	What to trust	What to do	Comparing & contrasting	Context‐specific	Closed‐response	Freely available
Critical thinking Assessment Test (CAT)^1^	X			STEM (broad)		
Lawson Test of Scientific Reasoning^2^	X			STEM (broad)		X
Test of Scientific Literacy Skills (TOSLS)^3^	X			STEM (broad)	X	X
Experimental Design Ability Test (EDAT)^4^	X	X		STEM (broad)	X	X
Test of Critical Thinking in Biology (TCTB)^5^				Biology	X	
Biological Experimental Design Concept Inventory (BEDCI)^6^	X			Biology	X	X
Biology Science Quantitative Reasoning Exam (BioSQuaRE)^7^				Biology	X	X
Biological Variation in Experimental Design And Analysis (BioVEDA) assessment^8^	X			Biology	X	X
Concise Data Processing Assessment (CDPA)^9^				STEM (broad)	X	X
Data Handling Diagnostic (DHD)^10^				STEM (broad)	X	X
Neuron Assessment^11^	X			Cell biology		X
Rubric for Experimental Design (RED)^12^	X			Biology		X
*Proposed* Biology Lab Inventory of Critical Thinking in Ecology (Eco‐BLIC)	X	X	X	Biology/ Ecology	X	X

*Note*: Adapted from table 1 in Walsh et al. (2019).

1. Stein et al. (2006); 2. Lawson (1978); 3. Gormally et al. (2012); 4. Sirum and Humburg (2011); 5. McMurray et al. (1991); 6. Deane et al. (2014); 7. Stanhope et al. (2017); 8. Hicks et al. (2020); 9. Day and Bonn (2011); 10. Bates and Galloway (2010); 11. Dasgupta et al. (2016); 12. Dasgupta et al. (2014). Full citations listed under References.

Research also suggests that critical thinking is context‐ and domain‐specific (Pithers & Soden, 2000; Willingham, 2008). Thus, critical thinking assessments should be embedded in a domain or disciplinary context, such as ecology. To disentangle the assessment of critical thinking skills from the assessment of students' knowledge about the context, one strategy is for the disciplinary context of the assessment to be accessible, such that all content knowledge needed to effectively complete the questions is present and at an appropriate content‐level for participants (Schwartz et al., 2016). While some of the available STEM critical thinking instruments summarized in Table 1 are biology‐specific, none to date have an ecology‐specific context.

Other design considerations include the structure and availability of the questions themselves (Table 1). In particular, students can better critique experimental scenarios when asked to explicitly compare and contrast, as opposed to evaluating each in turn (Heim et al., 2022). Open‐response and closed‐response formats also likely elicit different forms or levels of critical thinking (Pate, 2012). For example, while open‐response questions may elicit more creativity in the exploration of concepts or topics, closed‐response questions allow for more focused comparisons between groups or ideas (Pate, 2012; Quinn et al., 2018). Additionally, closed‐response instruments better meet the need for large‐scale evaluation of ecology courses because they can be scored and analyzed more quickly than open‐response instruments. Finally, freely available instruments are more accessible to instructors than ones that require payment.

### 1.2 Purpose and research aims

The Biology Lab Inventory of Critical Thinking in Ecology (Eco‐BLIC) assesses students' critical thinking skills related to experimentation in ecology. Our goal was to create an assessment based on the design principles in Table 1: closed‐response compare and contrast questions, discipline‐specific, and freely available. This instrument would complement existing critical thinking assessments (Table 1) and provide a novel way to assess undergraduate student critical thinking in courses that include ecology. Using frameworks from Vision and Change (AAAS, 2011) and the Advancing Competencies in Experimentation–Biology (ACE Bio) Network (Pelaez et al., 2018), and building from a related instrument in physics, the Physics Lab Inventory of Critical Thinking (PLIC; Quinn et al., 2018; Walsh et al., 2018; Walsh et al., 2019), we created experimental scenarios and questions intended to probe students' critical thinking skills. The scenarios and questions were designed to assess a range of students from multiple institutions and were research‐validated following standard procedures, including comparing open‐ and closed‐response versions, interviewing students, administering the assessments in multiple institutional contexts, and getting feedback from experts (Adams & Wieman, 2011). In this article, we answer the following research questions:

1. What is the statistical evidence of validity and reliability for the Eco‐BLIC?
2. How do student and expert responses align when evaluating the two components of critical thinking (i.e., what to trust and what to do)?

## 2 METHODS

### 2.1 Question development & format

We developed Eco‐BLIC questions through an iterative and stepwise process aligned with the standards of instrument design (De Vellis, 2003); these stepwise processes are further described below. Others have used similar methods to design biology concept assessments (Bass et al., 2016; Couch et al., 2015; NRC, 2012; Smith et al., 2008; Table 2). The Eco‐BLIC development is similar to the approach used for the Physics Lab Inventory of Critical Thinking (PLIC; Walsh et al., 2019). The PLIC is a 10‐question, closed‐response assessment that presents the t experimental methods and findings from two hypothetical physics research groups, one which uses a simpler approach and the other which uses a more complex approach (Walsh et al., 2019) Both are testing the relationship between the period of oscillation of a mass hanging from a spring. The questions ask respondents to evaluate the data and methods and propose next steps for each group. The PLIC underwent similar development, validity, and reliability testing as those presented here for the Eco‐BLIC (see Walsh et al., 2019 for details).

**TABLE 2:** Overview of Eco‐BLIC development.

Identify common themes encountered in introductory undergraduate biology and ecology courses (e.g., predator–prey relationships) and conduct literature review to ensure content intended to be included in scenarios is scientifically accurate.
2 Draft two sets of scenarios asking students to evaluate each research group, predict next steps, and compare between research groups.
3 Iteratively modify questions based on: Results from administering Eco‐BLIC to students: ○ Open‐response version (Fall 2019 & Spring 2020): 336 responses at one institution ▪ Student think‐aloud interviews for open‐response version (Spring 2020): 12 students at one institution ○ Draft closed‐response version (Fall 2020: 711 responses at 10 institutions; Spring 2021: 901 responses at 8 institutions) ▪ Student think‐aloud interviews for closed‐response version (Spring 2021): 21 students at one institution Feedback from 20 experts across at least* 7 institutions (Spring 2021) regarding the accuracy and clarity of each question (*some experts preferred not to identify their institution)
4 39 faculties from 29 institutions took the Eco‐BLIC to determine the scoring scheme. They were also given the opportunity to comment on any issues with scientific accuracy and clarity (Summer 2021)
5 Administer final version of Eco‐BLIC (Fall 2021 & Spring 2022): 1103 responses from introductory and advanced students at 16 institutions *n* = 179 matched pre‐/post‐tests for Fall 2021 *n* = 222 matched pre‐/post‐tests for Spring 2022 *n* = 401 matched pre‐/post‐tests total
6 Analyze student performance overall for each question (percent correct), difficulty and discrimination for each question, and evidence of reliability (Cronbach's alpha and item‐test correlations)

The Eco‐BLIC is administered via Qualtrics and provides students with experimental scenarios in which they learn about how different researchers approach answering the same question about feeding behaviors in a specific predator–prey relationship (Appendix A). Predator–prey relationships are commonly encountered in high school and introductory biology and ecology courses (Ginovart, 2014; Wasson, 2021) and often employ easy‐to‐analyze organism count data, thus making the content in the Eco‐BLIC broadly accessible.

Students engage with two scenarios. One scenario is based on relationships between smallmouth bass (*Micropterus dolomieu*) and comb‐mouthed minnow mayflies (*Ameletus cryptostimulus*), while the second is based on great‐horned owls (*Bubo virginianus*) and house mice (*Mus musculus*). In the bass‐mayfly scenario, students explore whether smallmouth bass selectively feed on larger or smaller mayflies. In the owl‐mouse scenario, students explore how the presence of a great‐horned owl influences the amount of time that mice spend feeding. As the Eco‐BLIC is intended to measure critical thinking, students are not required to have extensive content knowledge beyond the information that is provided in the scenario prompts. Although the scenarios are presented across multiple pages, students may go back to earlier pages in order to limit cognitive load.

Within each scenario, there are two research groups—one conducts their study in a laboratory‐based setting, while the other conducts their study in a field‐based setting. The descriptive prompts for each research group include a figure showing data, from which students are expected to form hypotheses and draw conclusions. There is a multiple‐choice prompt comprehension question asking students to interpret a figure (Table 3) and an open‐response question asking students to explain their reasoning for their initial hypothesis. Students are later asked to compare the experiments in these two distinct settings, lab versus field scenarios. There is not one perfect and one problematic research group, as each group's study has both strong and weak features.

**TABLE 3:** Examples of question formats, types, and examples used in the Eco‐BLIC.

Format	Type	Element of critical thinking measured	Eco‐BLIC (owl‐mouse scenario, lab group)
Multiple choice	Prompt comprehension question Response type: *Single choice closed‐response* *Not included in final scoring scheme*	Guides thinking about what to trust and what to do next	What do you think Group 1 should say about the feeding behavior of mice while great‐horned owl calls play? Mice spend less time at the food bowl in the presence of an owl predator call. Mice spend more time at the food bowl in the presence of an owl predator call. Mice spend the same amount of time as they usually do at the food bowl in the presence of an owl predator call. There is not enough evidence to determine mouse feeding behavior.
Multiple choice	Research group comparison items Response type: *Single choice closed‐response*	What to trust	How do you think Group 1 and Group 2 performed in the following categories? *Used an appropriate duration of time for the study (Group 1: one night; Group 2: two nights)* Group 1 was more effective Group 2 was more effective Both groups were highly effective Both groups were minimally effective
Multiple response	Next steps items Response type: *Up to 3 multiple responses can be selected* *For brevity, only 5 of the possible 13 options for this question are presented in the column to the right	What to do	What should Group 1 do next? (Select up to 3 options total) Redesign the study to run for a longer period of time Show a visual of an owl while owl calls play Increase the number of mice in the study Account for human error Conduct statistical analyses

*Note*: These examples are from the owl‐mouse scenario, and this table was adapted from table 2 in Heim et al. (2022).

The two primary types of scored questions included in the Eco‐BLIC are research group comparison items, “what to trust” (which ask students to compare study features between the lab and field experiments) and next steps items, “what to do” (which ask students to evaluate next steps for both the lab and field studies within an experimental scenario). Questions are presented in multiple formats, including multiple‐choice questions and multiple response questions in which students are asked to choose up to three responses from a list of 11 or 13 options (examples in Table 3).

At the end of the instrument, students are asked to complete a short demographic survey, including questions about race/ethnicity, gender, major, and prior research experience (Appendix A).

### 2.2 Participants and institutions

We administered versions of the Eco‐BLIC to students from a diverse range of institutions (Table 4). We recruited participants mainly through professional organization listservs and focused emails to potentially interested instructors; we only required that participating courses focus on “ecology concepts and topics.” Approximately 30% of students were first years, 18% were sophomores, 24% were juniors, and 28% were seniors. Nearly 66% had declared a major in biology or another life science. Over 60% of participating students identified as women and most students identified as White (56%), Hispanic or Latinx (19%), or Asian (17%). Most participants were recruited from general ecology (56%) and general biology (13%) courses, though the remaining 30% of participating courses covered broad topics (i.e., introductory courses in evolution and integrative biology and chemistry, and advanced courses in field biology, ecology, aquatic biology, botany, and ornithology). The majority of participating students were from introductory courses (91%), while 9% were from advanced courses. Participating courses had enrollment sizes ranging from approximately 10–350 students, with an average enrollment of approximately 100 (enrollment changes throughout the duration of courses limited our ability to report on exact enrollment counts).

**TABLE 4:** Summary of institutions that participated in administering draft (above horizontal line) and final (below horizontal line) versions of the Eco‐BLIC.

Version	Research activity	# institutions	Total # of student responses*	Total # of courses
Draft	Doctoral Universities: Very High Research Activity	5	1293	15
Draft	Doctoral/Professional Universities	1	168	5
Draft	Master's Colleges & Universities: Larger Programs	2	212	5
Draft	Baccalaureate Colleges: Arts & Sciences Focus	2	77	2
Draft	Associate's Colleges: High Transfer‐High Traditional	1	208	2
Final	Doctoral Universities: Very High Research Activity	6	838 (264)	12
Final	Doctoral/Professional Universities	3	67 (26)	3
Final	Master's Colleges & Universities: Larger Programs	3	135 (43)	3
Final	Master's Colleges & Universities: Small Programs	1	23 (9)	1
Final	Baccalaureate Colleges: Arts & Sciences Focus	2	138 (61)	2
Final	Canadian Institution (does not have Carnegie classification)	1	2 (0)	1

*Note*: Research activity information based on Carnegie classifications.

* Values in parentheses indicate the total number of pre‐ and post‐test matched individual data we collected from each institution type for the final version. The lower response rate from Doctoral Universities: Very High Research Activity on the final version was largely due to instructors of participating courses not offering extra credit for completing the Eco‐BLIC.

### 2.3 Open‐response version (Fall 2019 and Spring 2020)

The open‐response version of the Eco‐BLIC included open‐response questions to gather student thinking in their own words. Similar to the instrument development process used for other undergraduate assessments, questions were iteratively revised for clarity, length, and scientific accuracy based on written responses from students (Adams & Wieman, 2011; Bass et al., 2016; Couch et al., 2015; NRC, 2012; Smith et al., 2008; Walsh et al., 2019).

We also conducted student think‐aloud interviews to achieve cognitive validation because they are an effective way to provide “evidence that survey items are interpreted by participants in the same way the researcher intended before the instrument is administered to a large sample” (p. 2, Trenor et al., 2011). We recruited 12 introductory and advanced undergraduates in Spring 2020 for semistructured think‐aloud video‐ and audio‐recorded interviews via Zoom (Marbach‐Ad et al., 2009; Smith et al., 2008). Students were asked to think aloud and explain their reasoning as well as any points of confusion, and the results were used to inform improvements to the language, structure, and clarity of the instrument (Anders & Simon, 1980; Marbach‐Ad et al., 2009; Smith et al., 2008). We generated the closed‐response version of the Eco‐BLIC in the same manner as the PLIC (see Walsh et al., 2019 for more detail regarding this process). For example, in developing the closed‐response Eco‐BLIC, we adopted similar question formats as the PLIC including using multiple and single response items (Table 3). We also incorporated students' wording from open response questions in creating the closed‐response questions rather than introducing expert jargon or terminology for ease of comprehension.

### 2.4 Draft closed‐response version (Fall 2020, Spring 2021, & Summer 2021)

We developed and iteratively revised the closed‐response version of the Eco‐BLIC during the 2020–2021 academic year (Table 2). Students completed one version of the assessment on the pretest and were randomly assigned one of two versions for the post‐test (approximately the same number of students took each version). To explore whether the ordering of scenario prompts had any influence on how students responded to questions on the Eco‐BLIC, version #1 introduced the field‐based research groups before the lab‐based research groups in each scenario, while version #2 introduced the lab‐based research groups before the field‐based research groups in each scenario. We found no significant difference (*t*‐tests and ANOVA) in how students responded to questions when the ordering of scenarios was changed. Based on the results, we maintained the original question ordering (i.e., field‐based followed by lab‐based for the bass‐mayfly scenario and lab‐based followed by field‐based for the owl‐mouse scenario) in subsequent versions. We also iteratively used the feedback we received on these draft versions of the Eco‐BLIC to clarify instructions and wording, add in missing elements, or remove questions and/or responses that were deemed unnecessary.

In a later draft version administered in Spring 2021, we also explored how students evaluated the quality of data in lab and field studies if individual evaluation questions were provided (i.e., questions that ask students to evaluate the strengths and weaknesses of different study features for each research group in a scenario individually). Ultimately, we found that students did not answer questions on the Eco‐BLIC differently when the individual evaluation questions were present, and thus, we removed these questions in subsequent versions of the assessment (Heim et al., 2022).

Next, we conducted semistructured think‐aloud interviews to explore question clarity on the revised assessment (Table 2). We used the same methods to achieve cognitive validation of the closed‐response version as we did the open‐response version. Student participants spanned a range of biology concentrations (e.g., ecology and evolution, neurobiology, and physiology).

In Spring 2021, 20 experts provided feedback on the draft closed‐response version of the Eco‐BLIC. The experts were recruited through professional organization listservs and included biology and ecology professors, instructors/lecturers, and postdoctoral associates, from a wide array of institutions (e.g., 4‐year institutions and community colleges). Experts were asked to both respond to the questions in the Eco‐BLIC and offer written feedback on each page of the assessment (e.g., to note if wording was unclear, content was scientifically inaccurate, or the assessment duration was too long). We used this feedback to develop the final version of the Eco‐BLIC.

### 2.5 Final version of the Eco‐BLIC (Fall 2021 and Spring 2022)

#### 2.5.1 Administration

We administered the final version of the Eco‐BLIC to undergraduates across a range of institution types to confirm the utility of our instrument (Table 4). We sent participating instructors a survey link (Qualtrics, Provo, UT) to share with their students through course announcements, emails, and/or learning management systems and recommended that instructors provide credit for completing the Eco‐BLIC to incentivize students. In general, students took between 20 and 30 min to complete the Eco‐BLIC, which is administered online. Instructors assign the pre‐Eco‐BLIC to their students in the first 2 weeks of a course and the post‐Eco‐BLIC in the last 2 weeks of a course, either as an in‐class or out‐of‐class assignment. Students are not informed of their pre‐ and post‐test scores. Prior to conducting any statistical analyses, we excluded data in which students did not consent to have their responses used for research, were not 18 years of age or older, did not include their name (for pre‐post test matching), and/or completed the assessment in less than 5 min.

#### 2.5.2 Scoring scheme

The Eco‐BLIC scoring scheme was based on responses from 39 expert biologists (Table 2). The experts were recruited mainly through professional organization listservs and included biology and ecology professors, instructors/lecturers, and postdoctoral associates, from a wide array of institutions (e.g., 4‐year institutions and community colleges). Experts were asked to respond to the Eco‐BLIC questions and also given the opportunity to offer written feedback on each page of the assessment (e.g., to note if wording was unclear). The suggestions from the experts were minimal, and we only made small wording adjustments based on their suggestions.

We adapted the Eco‐BLIC scoring scheme from the scoring scheme developed for the PLIC (Walsh et al., 2019). Because expert responses suggested that there was no single correct answer for scored questions on the Eco‐BLIC, an all‐or‐nothing scoring scheme (in which students would receive full credit for choosing a single correct response or no credit for choosing alternate responses) for scored questions would be inaccurate. Instead, the fraction of experts selecting each response served as an estimate of the relative correctness of each response choice.

##### Prompt comprehension questions

The multiple‐choice prompt comprehension questions (Table 3) and an open‐response question asking students to explain their reasoning for their initial hypothesis are not scored. These questions are to help orient students to the experimental scenario.

##### Research group comparison items

**FIGURE 1:**
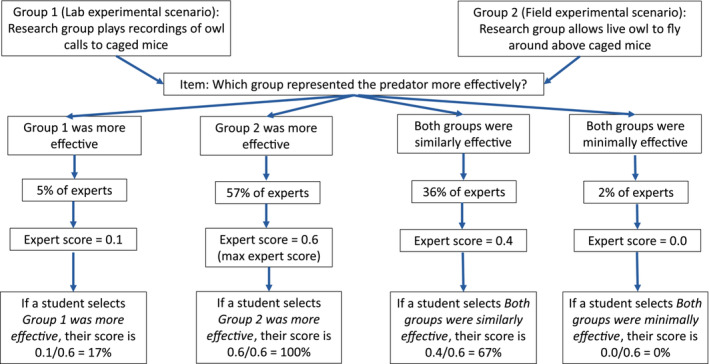
Sample scoring scheme for research group comparison questions. Data shown is for the item “Represented the predator appropriately.”

Research group comparison items (Table 3), an indicator of “what to trust” in our assessment, are included in the scoring scheme. All research group comparison items on the Eco‐BLIC have a multiple‐choice format, in which students can choose a single option from four possible responses. We assign values to each item based on the fraction of experts who chose that item out of the total number of experts who responded to that item. For example, Figure 1 shows the calculation when comparing the lab and field studies in the owl‐mouse scenario for the *Represented the predator appropriately* item. We then added the scores for each item within the scenario to get the owl‐mouse research group comparison score. We apply the same scoring scheme to the bass‐mayfly research group comparison questions.

##### Next steps items

Next steps items (the scored indicator of “what to do” in our assessment) are included in the scoring scheme as well. All next steps items on the Eco‐BLIC have a multiple response format, in which students can choose up to three options from a list of responses (Table 3). We assign values to each item based on the fraction of experts who chose that item out of the total number of responses we received for that single question (rounded to the nearest tenth). For example, when evaluating next steps for the field study in the owl‐mouse scenario, students are asked to choose up to three responses from a list of 13 items. To account for the fact that students can choose anywhere from one to three responses for each next step question, we use a similar approach as the PLIC, in which the sum of the total value of responses selected is divided by the maximum value of the number of responses selected (see Walsh et al., 2019 for further details). Using this scoring scheme for next steps questions:

*V*_max1_ = the most commonly selected item among experts.
*V*_max2_ = (*V*_max1_) + (the second most commonly selected item among experts).
*V*_max3_ = (*V*_max1_) + (*V*_max2_) + (the third most commonly selected item among experts).

Thus, if a student selects one, two, or three responses, they will score the maximum number of points if they select the one, two, or three highest valued responses, respectively. For example, when evaluating next steps for the field study in the owl‐mouse scenario, 73% of experts reported that *conducting statistical analyses* was most important, followed by *sampling mice from other fields* (41%) and *repeating the study to gather more data* (30%). These percentages would translate to scores of 0.7, 0.4, and 0.3, respectively. In this example, using the *V*_max_ equations outlined above, the maximum scores for choosing one, two, and three items are, respectively:

*V*_max1_ = 0.7.
*V*_max2_ = 0.7 + 0.4 = 1.1.
*V*_max3_ = 0.7 + 0.4 + 0.3 = 1.4.

A student who chooses only one option would need to select *conducting statistical analyses* (the top expert response) to receive a maximum score on this next steps question, while a student who chooses only two next steps items would need to select *conducting statistical analyses* and *sampling mice from other fields* (the top two expert responses) to receive a maximum score on this question. In a case where a student selects one of the top expert responses (e.g., *conducting statistical analyses*, *V*_max1_) and one of the nonexpert responses outside of *V*_max1–3_ (e.g., *account for human error*, chosen by 0% of experts), the student would receive points for choosing *V*_max1_ and would receive no points for choosing the nonexpert response. Therefore, students are not disproportionately penalized for selecting more or fewer responses on next steps questions using this scoring scheme. To normalize the next steps scores, we divide students' score by the total maximum expert score for the same number of selected responses (i.e., *V*_max_).

##### Total Eco‐BLIC score

The student's total score on the Eco‐BLIC is obtained by summing the research group comparison scores (two subscores, one for the research group comparison items in the bass‐mayfly scenario and one for the research group comparison items in the owl‐mouse scenario) and the next steps question scores (four subscores, one for each lab and field group in the bass‐mayfly and owl‐mouse scenarios). Because there are only two subscores for research group comparison items compared with four for next steps questions, we multiplied the weight of each research group comparison subscore by two. Therefore, the maximum attainable score on the Eco‐BLIC is eight points (four points from the research group comparison questions and four points from the next steps questions). While our scoring scheme is based on fractions of points, below we report scores as percentages for comparison purposes.

All statistical comparisons discussed below (i.e., when a *p*‐value is reported) are based on either *t*‐tests (for comparisons of two groups) or ANOVAs (for comparisons of more than two groups).

## 3 FINDINGS

Below, we describe findings from different analyses investigating the reliability of the Eco‐BLIC, including test and question difficulty, question discrimination, internal consistency and question‐test correlations, test–retest reliability, and concurrent validity. Interquartile range is abbreviated as IQR. Note that we report scores as percentages for comparison purposes.

### 3.1 Test and question difficulty

**FIGURE 2:**
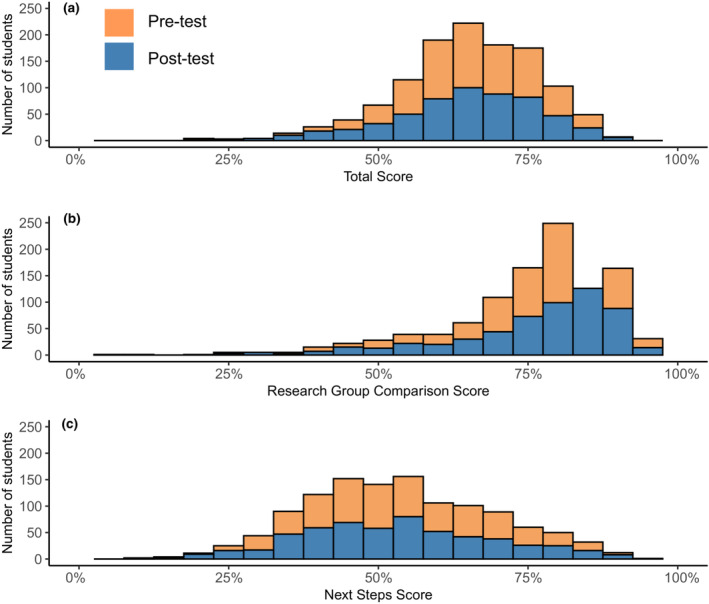
Distributions of students' (a) total scores, (b) research group comparison scores, and (c) next steps scores (pretest: *n* = 638, post‐test: *n* = 564). Minimum and maximum scores ranged from (a) 0%–88% on the pretest and 20%–91% on the post‐test; (b) 0%–97% on the pretest and 7%–98% on the post‐test; and (c) 0%–91% on the pretest and 17%–93% on the post‐test. Medians and IQRs were: (a) pretest: median = 65%, IQR = 14%, post‐test: median = 66%, IQR = 16%; (b) pretest: median = 80%, IQR = 13%, post‐test: median = 80%, IQR = 16%; (c) pretest: median = 53%, IQR = 22%; post‐test: median = 53%, IQR = 23%.

The average total score on the final version of the Eco‐BLIC (*n* = 1103 student responses) was 65% for both the pretest and post‐test (Figure 2a). There was no significant difference in total scores among student responses (*p* > .05).

We also examined average total scores across question types. The average total score across the research group comparison questions was 77% on the pretest and 76% on the post‐test (Figure 2b). The average total score across next steps questions was 54% on the pretest and 53% on the post‐test (Figure 2c). Similar to the total score data, there was no significant difference in research group comparison or next steps scores among student responses on the pre‐ and post‐tests (*p* > .05), both for pooled responses and for matched data. While the research group comparison scores were negatively skewed (i.e., the distribution leans to the right), the total and next steps scores followed a more normal distribution. Thus, we report median and IQR along with means in our results and figure legends, to allow for more meaningful comparisons.

**FIGURE 3:**
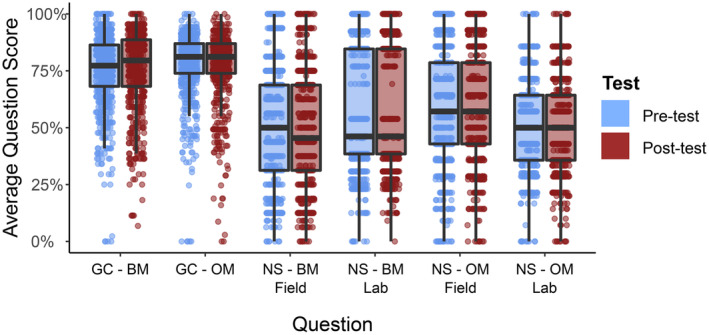
Median scores and distributions for each of the six primary Eco‐BLIC questions (representing item difficulty). Horizontal lines represent the median and lower and upper quartiles. GC = research group comparison, NS = next steps, BM = bass‐mayfly scenario, OM = owl‐mouse scenario.

The average score per question ranged from 50% to 78% on the pretest and from 50% to 77% on the post‐test (Figure 3), within the acceptable range noted in prior instrument validation studies (Ding & Beichner, 2009; Doran, 1980). We did not find any significant differences between item difficulty for each question between pre‐ and post‐test responses (*p* > .05). The average mean scores for the research group comparison questions were higher (75%–78%) than the average scores for the next steps questions (50%–58%; Figure 3). While we did not find significant differences between pre‐ and post‐test total, research group comparison, or next steps scores with the aggregate data (*n* = 1103 responses), we measured significant changes in pre‐ and postscores in three individual courses (two general and one advanced ecology courses).

### 3.2 Question discrimination

We used question‐test correlations (i.e., correlations between students' total scores on the Eco‐BLIC and their scores on individual questions) to explore how well each question discriminated between low‐ and high‐performing students. Question‐test correlations were greater than 0.47 for all pretest questions, greater than 0.40 for all post‐test questions, and greater than 0.44 for all compiled responses (i.e., pre‐ and post‐test responses combined, including unmatched responses; Table 5). All of these question‐test correlations are above the accepted value of 0.20 reported in the assessment literature (Ding & Beichner, 2009).

**TABLE 5:** Question‐test correlations as a measure of question discrimination.

	BM research group comparison	OM research group comparison	BM field next steps	BM lab next steps	OM lab next steps	OM field next steps
Pretest Data	0.67	0.66	0.54	0.57	0.47	0.59
Post‐test Data	0.71	0.67	0.53	0.59	0.40	0.62
Compiled Data	0.69	0.66	0.54	0.58	0.44	0.60

Abbreviations: BM, bass‐mayfly scenario; OM, owl‐mouse scenario.

### 3.3 Internal consistency

Based on our definition of critical thinking (what to trust and what to do), we hypothesized that the Eco‐BLIC would measure two distinct constructs. To explore this assertion, we conducted exploratory factor analysis (EFA) using student responses from Fall 2021 (i.e., the first set of responses on the final version of the Eco‐BLIC) and confirmatory factor analysis (CFA) using student responses from Spring 2022 (i.e., the second set of responses on the final version of the Eco‐BLIC). EFA is used to “explore the possible underlying factor structure of a set of observed variables without imposing a preconceived structure on the outcome” while CFA is used to “test the hypothesis that a relationship between observed variables and their underlying latent constructs exist” (p. 1, Suhr, 2006). We chose to conduct factor analysis in this way to first establish and define the constructs of our instrument without preconceived expectations and then to confirm that the patterns we found in our Fall 2021 dataset were sound.

We conducted the EFA with oblique rotation—or rotating the axes during factor analyses at an angle other than 90 degrees to improve the interpretation of factor loadings (Suhr, 2006)—as this adjustment does not assume independence of student responses. We found that questions loaded primarily onto two factors that cumulatively explained nearly 35% of the variance of students' scores on the six primary Eco‐BLIC questions. After analyzing factor loadings, we determined that the group comparison questions, aligned with what to trust, loaded primarily onto the first factor (loadings ranging from 0.35 to 0.55) while the next steps questions, aligned with what to do next, loaded primarily onto the second factor. The next steps questions for the owl‐mouse lab scenario loaded less strongly (0.08). One possible explanation comes from think‐aloud interviews with students. Students indicated that choosing next steps for the owl‐mouse lab scenario was particularly challenging because nearly all of the options seemed viable or practical to implement in a future lab experiment.

We then conducted CFA on Spring 2022 data by creating a model in which we input the first two research group comparison questions as the research group comparison factor and the four next steps questions as the next steps factor. The model fit measures were within reasonable parameters (RMSEA = 0.05, SRMR = 0.03; Hu & Bentler, 1999). The research group comparison questions loaded well onto the first factor (loadings ranging from 0.68 to 0.70), and the next steps questions loaded well onto the second factor (loadings ranging from 0.34 to 0.67). Thus, we found similar patterns using both analyses, though we cannot compare the EFA and CFA factor loadings on a 1:1 scale because EFA and CFA use different parameters.

### 3.4 Test–retest reliability

Test–retest reliability of an instrument is usually measured by having the same respondents complete the assessment multiple times under the same conditions. As longitudinal administration of the Eco‐BLIC is not plausible because students may gain knowledge and skills in their biology courses over time, we were not able to establish test–retest reliability using the same students' scores across semesters. Instead, we used pretest scores of students in the same courses across different semesters (Fall 2021 and Spring 2022) to estimate test–retest reliability. Since the instructors for each course remained consistent across the different semesters, we made the assumption that the general student sample was similar across semesters for each class. This approach for course‐level test–retest reliability was also used for the PLIC (Walsh et al., 2019).

We had two separate courses from two institutions complete the Eco‐BLIC in two different semesters (Fall 2021 and Spring 2022). As seen in Table 6, the pretest scores for both Class A and Class B were not significantly different between semesters.

**TABLE 6:** Summary of test–retest results comparing mean and median pretest scores across two consecutive semesters of the same course.

Class	Term	*N*	Pretest mean score	Pretest median score	*p*‐Value
Class A	Fall 2021	80	63%	64% (13%)	.98
	Spring 2022	102	64%	67% (13%)	
Class B	Fall 2021	19	59%	53% (16%)	.39
	Spring 2022	29	62%	60% (9%)	

*Note*: Classes A and B were introductory ecology courses at different institutions. Scores have been normalized out of one point. Values in parentheses next to each median score are interquartile ranges (IQR).

### 3.5 Concurrent validity

We analyzed two forms of concurrent validity—“a measure of the consistency of performance with expected results” (p. 10, Walsh et al., 2019)—for our instrument. First, we compared question scores on the pretest between students in introductory courses (*n* = 582), students in advanced courses (*n* = 55), and experts (*n* = 39), with the expectation that experts would have higher scores than students. We found that the total scores between introductory (mean = 65%, median = 65%, IQR = 15%) and advanced (mean = 66%, median = 65%, IQR = 14%) students were not significantly different (*p* = .66). Total scores between introductory students and experts (mean = 78%, median = 80%, IQR = 10%; *p* < .001) and between advanced students and experts (*p* < .001) were significantly different.

**FIGURE 4:**
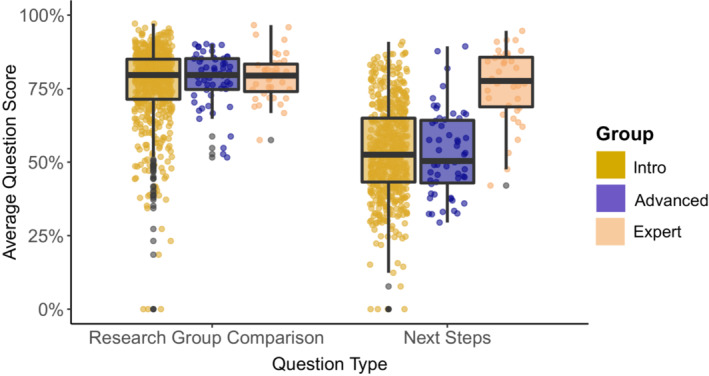
Research group comparison and next steps scores for introductory students (*n* = 582), advanced students (*n* = 55), and experts (*n* = 39). Horizontal lines represent the median and lower and upper quartiles, while dots indicate outliers. Research group comparison scores: introductory students (mean = 76%, median = 80%, IQR = 14%), advanced students (mean = 78%, median = 80%, IQR = 10%), and experts (mean = 79%, median = 79%, IQR = 9%). Next steps scores: introductory students (mean = 54%, median = 53%, IQR = 22%), advanced students (mean = 53%, median = 50%, IQR = 21%), experts (mean = 76%, median = 78%, IQR = 17%).

To further parse out these patterns, we explored differences in research group comparison scores and next steps scores between these three groups. When comparing the research group comparison scores between introductory students, advanced students, and experts, there were no significant differences between groups (*p* > .05; Figure 4). We also found that while introductory and advanced students' next steps scores did not differ (*p* = .75), experts' next steps scores were significantly higher than those of introductory students (*p* < .001) and advanced students (*p* < .001; Figure 4).

The second form of concurrent validity analyzed how scores differ based on students' prior research experience. We expected that students with more research experience would be more likely to have higher scores on the Eco‐BLIC because of their familiarity with the scientific process. We compared question scores on the pretests between students with no research experience (*n* = 454) and some research experience (i.e., one or more terms; *n* = 179). In the question, we defined a term as a semester, quarter, or summer session, and the survey indicated that research experiences should have been supervised by a faculty mentor.

**FIGURE 5:**
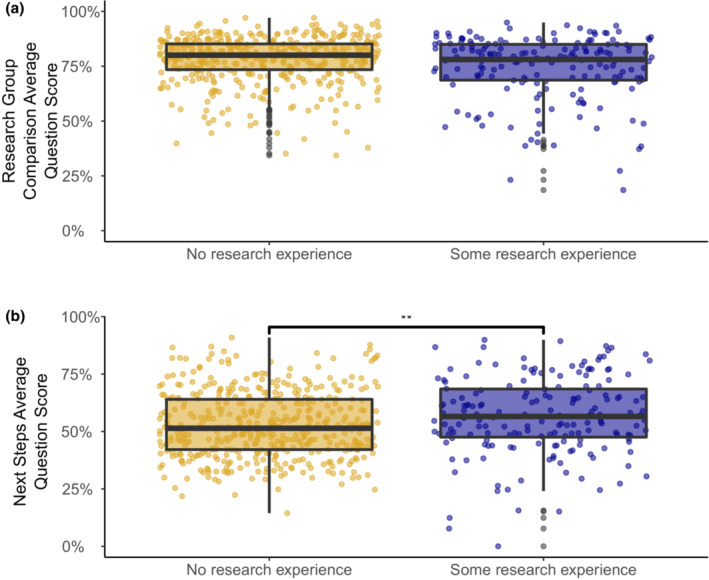
Scores separated by the amount of student research experience for (a) research group comparison questions and (b) next steps questions. *Some* signifies one or more terms of research experience. Horizontal lines represent the median and lower and upper quartiles, while dots indicate outliers. Next steps question scores for students with no research experience (mean = 53%, median = 51%, IQR = 22%, *n* = 454) compared with some research experience (mean = 57%, median = 57%, IQR = 21%, *n* = 179). **Indicates significance (*p* < .001).

We did not find any differences in research group comparison scores across students with varying research experience (Figure 5a). However, we found that next steps scores were significantly higher for students with some research experience than for students with no research experience (*p* = .0006; Figure 5b).

## 4 DISCUSSION

Results from tests of validity and reliability indicate that we have successfully developed a new instrument—the Eco‐BLIC—that assesses students' critical thinking skills related to experimentation in ecology. The instrument has utility across different instructional settings and institution types. Our intention is for instructors to use the Eco‐BLIC as a measure of students' critical thinking over the duration of a course. Researchers can use the tool to further evaluate instructional strategies that support the development of students' critical thinking skills in ecology. From the validation results, we identified consistent trends regarding these skills, which we discuss below.

### 4.1 Students' Eco‐BLIC scores did not change over time

The overall lack of change from pre‐ to postscores across a range of biology and ecology courses in our study emphasizes a possible misalignment in student outcomes and learning activities and assessments in the classroom. Further, this observation suggests that, although ranked as one of the most important and necessary outcomes of undergraduate degree programs (Gencer & Dogan, 2020; Murawski, 2014), critical thinking about experiments may not be commonly developed in the biology and ecology classroom (Fox & Hackerman, 2003; Handelsman, 2004). Bissell and Lemons (2006) attribute the challenges of incorporating pedagogical techniques that aim to improve critical thinking skills to (1) a lack of one common definition of critical thinking and (2) a limited number of instruments available to measure and assess critical thinking in the classroom. We encourage instructors to leverage the Eco‐BLIC as a tool for measuring and assessing their critical thinking to better align instruction with critical thinking learning outcomes. However, we also note that we did measure significant increases in pre‐ to postscores on the Eco‐BLIC in three participating courses (with critical thinking gains in several other courses approaching significance), which suggests that the Eco‐BLIC can measure changes in students' critical thinking in individual courses with unique instructors. An important next step is to describe the learning activities, assessments, and learning objectives for each course and instructor to better understand alignment of these classroom aspects with critical thinking. Additionally, integrating similar critical thinking instructional activities across participating courses and giving the Eco‐BLIC pre and post could shed light on what instructional components influence critical thinking gains in undergraduate biology.

As further evidence of the lack of development in critical thinking over time, we also found that introductory and advanced students did not differ in their Eco‐BLIC scores (Figure 4). Though it may seem intuitive that students in advanced courses would have more critical thinking skills than students in introductory courses and thus exhibit more expert‐like thinking when evaluating what to trust and what to do, this was not the case. Quitadamo and Kurtz (2007) also noted the disconnect between faculty expectations of senior undergraduates' critical thinking and their students' performance on critical thinking assessments (AACU, 2005). If students in introductory biology and ecology courses are not gaining critical thinking skills, and instructors of advanced courses are assuming that students already gained these skills earlier, the opportunity to actually gain these skills may never have occurred.

### 4.2 Students demonstrate less expert‐like thinking when deciding what to do

We consistently observed that while students think similarly to experts in evaluating what to trust (i.e., the research group comparison questions), students' responses were less expert‐like when deciding what to do (i.e., next steps questions; Figure 4). Walsh et al. (2019) found a similar pattern in physics scenarios using the PLIC, suggesting this result is not unique to ecology. Notably, students with at least one term of research experience scored significantly higher on the next steps questions than students who reported having no research experience (Figure 5b). This result supports our hypothesis that students with more research experience would have higher scores on the Eco‐BLIC because of their familiarity with the scientific process. If students have more authentic experience in making decisions about what to do next in their research (e.g., troubleshooting and proposing future directions for their project), it seems reasonable that they would be more likely to apply those skills on the Eco‐BLIC and thus score higher on the next steps questions compared with students with no research experience.

The discrepancy in students' abilities to decide what to do next could potentially be ameliorated by engaging students in undergraduate research opportunities to enhance critical thinking skills (Juanda, 2022). Gaining critical thinking skills has frequently been reported as a primary benefit for students participating in undergraduate research experiences (Bhattacharyya et al., 2018; Helix et al., 2022; Seifan et al., 2022; Seymour et al., 2004). Now that the Eco‐BLIC is available to assess students' critical thinking and students with research experience show more expert‐like thinking in evaluating what to do next, we should explore how to bring these skills to all students in our courses.

While we found that faculty‐mentored research experiences are helping students to gain necessary critical thinking skills (Figure 5), it is not practical for all undergraduates to partake in research experiences led by faculty (Wei & Woodin, 2011). One option is to bring inquiry and discovery lab‐based experiences to the classroom through course‐based undergraduate research experiences (CUREs), which have been found to encourage students' critical thinking–specifically their evaluation of what to do next in experimental scenarios (Brownell et al., 2015). CUREs also provide opportunities for students to think like expert scientists (Brownell & Kloser, 2015) and may promote iteration and thinking about next steps in the face of research failures (Gin et al., 2018), which is important to consider given that ecological data can be messy and unpredictable. Future work should disentangle whether students who take courses with opportunities to design their own authentic experiments see comparable gains in scores on the next steps items as students engaging in undergraduate research.

In addition to lab experiences, learning experiences in the lecture setting are often students' first exposure to foundational ecological concepts. In this setting, instructors have an opportunity to introduce authentic data and experimental design that may be unique from or complementary to the skills students are gaining in lab‐based course components. For example, instructors could implement case studies focused on analysis of ecological experiments or scenarios (e.g., American Museum of Natural History in their Ecology Disrupted Curriculum section: https://www.amnh.org/learn‐teach/curriculum‐collections/ecology‐disrupted/additional‐case‐studies; Carlin, 2019). Peer‐reviewed, evidence‐based teaching lessons in biology and ecology, with a focus on students' abilities to apply the process of science, also are available through the open‐education resource journal *CourseSource* (https://qubeshub.org/community/groups/coursesource) and Teaching Issues and Experiments in Ecology (TIEE) (https://tiee.esa.org/). The results of our analysis indicate that evaluation of these cases and lessons should include opportunities for students to answer questions about what researchers should do next. Further, instructors can now assess the effectiveness of such activities in enhancing their students' critical thinking skills over time using the Eco‐BLIC.

### 4.3 Limitations

To make this instrument easy for instructors to implement and score, we used a closed‐response format. However, this format can be limiting. For example, we are not able to glean students' reasons for choosing what to do and what to trust as we would with an open‐response format. To mitigate this limitation, the instrument validation process began with an open‐response version and included think‐aloud interviews with students to improve content validity and better understand their critical thinking processes. Given the number of options presented, students may have also experienced more cognitive load on the next steps questions versus the research group comparison questions. To mitigate cognitive load, we allowed students to return to the Eco‐BLIC scenario prompts and included next steps and research group comparison questions on separate pages in Qualtrics.

### 4.4 Conclusions

We collected validity and reliability evidence for the Eco‐BLIC, which demonstrates that it can be used to measure critical thinking across a range of biology and ecology courses to better understand how students evaluate both what to trust and what to do. Through assessing concurrent validity, we found that students demonstrate less expert‐like thinking when deciding what to do and that students in introductory and advanced courses do not differ in their critical thinking skills. Further, while students' Eco‐BLIC scores did not change over time, students with some amount of research experience had more expert‐like thinking on next steps questions compared with students who had no experience. The results indicate that instructors may wish to reflect on the alignment of their critical thinking‐related course learning outcomes and activities, deliberately design or adapt course materials to provide opportunities for students to gain critical thinking skills—particularly those focused on evaluating what to do next—and measure their students' critical thinking using instruments like the Eco‐BLIC. Currently, instructors of participating courses receive a summary report of their students' Eco‐BLIC scores from the research team to interpret critical thinking changes across the duration of their courses. In the future, the Eco‐BLIC will have an automated scoring system that will give instructors access to a more detailed breakdown of the scoring. Instructors interested in administering the Eco‐BLIC in an upcoming course can contact the corresponding author.

## AUTHOR CONTRIBUTIONS

**Ashley B. Heim:** Conceptualization (equal); data curation (equal); formal analysis (equal); investigation (equal); methodology (equal); validation (equal); visualization (equal); writing – original draft (equal); writing – review and editing (equal). **David Esparza:** Conceptualization (equal); data curation (equal); formal analysis (equal); funding acquisition (equal); investigation (equal); methodology (equal); validation (equal); visualization (equal); writing – review and editing (equal). **Natasha G. Holmes:** Conceptualization (equal); funding acquisition (equal); methodology (equal); supervision (equal); validation (equal); writing – review and editing (equal). **Michelle K. Smith:** Conceptualization (equal); funding acquisition (equal); methodology (equal); resources (equal); supervision (equal); validation (equal); writing – review and editing (equal).

## FUNDING INFORMATION

This work was supported by a National Science Foundation grant (DUE‐1909602) and a National Science Foundation Graduate Research Fellowship (DGE‐2139899). The funders had no role in study design, data collection and analysis, decision to publish, or preparation of the manuscript.

## CONFLICT OF INTEREST STATEMENT

The authors have declared that no competing interests exist.

## Supporting information


Appendix

## Data Availability

The datasets referenced in this article are not readily available because the approved study protocol and consent form explicitly state that this sensitive human subject data will be confidentially protected and will not be shared publicly due to the personal nature of the reflections. Requests to access additional information contained within the private datasets should be directed to corresponding author Ashley B. Heim, Ph.D. (abh229@cornell.edu) at the Department of Ecology and Evolutionary Biology at Cornell University.

## APPENDIX A

### A.1 Eco‐BLIC

The following questions will ask you about case studies where groups are exploring ecological environments. The aggregate results will be used to help develop the content for your biology course.

Please do NOT use outside resources to answer the questions.

[Insert consent form here]

In this part of the survey, you will read information and answer questions about two studies depicting a predator–prey relationship between smallmouth bass and mayflies. Both groups are conducting independent investigations. You can go back to previous pages of the survey using the red “back” arrow on the bottom of the page, if needed. Two groups of biologists are studying smallmouth bass (Micropterus dolomieu) and combmouthed minnow mayflies (Ameletus cryptostimulus; hereafter referred to as mayflies). Smallmouth bass eat young mayflies, which live at the water surface. Mayflies do not grow bigger than what smallmouth bass can eat. Both groups of biologists want to know whether smallmouth bass selectively feed on larger or smaller mayflies.

### A.2 Group 1's Study

We conducted surveys of nearby 30 ponds: 15 ponds that contained smallmouth bass and 15 ponds that contained no smallmouth bass.

*Below you will find a picture of one of Group 1's study sites*.

We measured 10 young mayflies within each pond and calculated the mean length of mayflies for each. We found the following pattern:

*Reminder: Each circle equals the mean (average) mayfly length per pond*.

What do you think Group 1 should say about the feeding pattern between smallmouth bass and mayflies?

Smallmouth bass selectively consume smaller mayflies.
Smallmouth bass selectively consume larger mayflies.
Smallmouth bass consume mayflies with no size preference.
There is not enough evidence to determine the feeding pattern.

Please explain your reasoning in the space below.

________________________________________________________________

How effective was Group 1, overall, in testing whether smallmouth bass selectively feed on larger or smaller mayflies?

Ineffective (1)
2
3
Effective (4)

What should Group 1 **do next**? (S*elect up to 3 options total.)*

Redesign the study to run for a longer period of time
Repeat the study using organisms from a randomly selected set of ponds
Control for biological variables (i.e., biotic factors)
Account for human error
Redesign the study for a controlled laboratory environment
Sample from more ponds
Conduct statistical analyses
Run a study where a variable is manipulated
Repeat the study to gather more data
Control for non‐biological variables (i.e., abiotic factors)
Other (Please describe in the box): __________________________________________________

Below is information about the second group of biologists studying smallmouth bass and mayflies. As a reminder, smallmouth bass eat young mayflies, which live at the water surface. Mayflies do not grow bigger than what smallmouth bass can eat. Both groups of biologists want to know whether smallmouth bass selectively feed on larger or smaller mayflies.

### A.3 Group 2's study

We collected smallmouth bass and young mayflies from a single pond. We established ten tanks and placed 100 mayflies in each tank. We placed one smallmouth bass in each tank for 24 h. The tanks are covered with a net, so the mayflies cannot move to different tanks.

*Below is a picture of Group 2's laboratory setup*.

We took a random sample of 20 mayflies from each tank before and after the 24‐hour period. We calculated the mean (average) length of mayflies in each sample. We found the following pattern:

*Reminder: Each circle equals the mean (average) mayfly length per tank. Matched samples are connected with a solid line*.

What do you think Group 2 should say about the feeding pattern between smallmouth bass and mayflies?

Smallmouth bass selectively consume smaller mayflies.
Smallmouth bass selectively consume larger mayflies.
Smallmouth bass consume mayflies with no size preference.
There is not enough evidence to determine the feeding pattern.

Please explain your reasoning in the space below.

________________________________________________________

How effective was Group 2, overall, in testing whether smallmouth bass selectively feed on larger or smaller mayflies?

Ineffective (1)
2
3
Effective (4)

What should Group 2 **do next**? (S*elect up to 3 options total.)*

Redesign the study to run for a longer period of time
Increase the number of tanks used in the laboratory
Control for other biological variables (i.e., biotic factors)
Account for human error
Redesign the study for an outdoor setting
Sample from more ponds
Conduct statistical analyses
Run a study where a variable is manipulated
Repeat the study to gather more data
Control for non‐biological variables (i.e., abiotic factors)
Other (Please describe in the box):

_________________________________________________

Which group do you think gained a more accurate understanding of the feeding pattern between smallmouth bass and mayflies?

Group 1
Group 2
Both groups gained an accurate understanding
Neither group gained an accurate understanding

How do you think **Group 1** and **Group 2** performed in the following categories?

**Table:**

	Group 1 was more effective	Group 2 was more effective	Both Groups were effective	Neither group was effective
Used an appropriate study setting (**Group 1:** ponds; **Group 2:** tanks)				
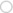
Used an appropriate study setup (**Group 1:** Ponds with and without bass; **Group 2:** Tanks before and after bass)				
Used appropriate methods to collect data (e.g., measuring mayfly length)				
Selected a sufficient sample size (**Group 1:** 10 mayflies per pond; **Group 2:** 20 mayflies per tank)				

How do you think **Group 1** and **Group 2** performed in the following categories?

**Table:**

	Group 1 was more effective	Group 2 was more effective	Both Groups were effective	Neither group was effective
Sampled from an appropriate number of ponds (**Group 1:** 30 ponds; **Group 2:** 1 pond)				
Ran appropriate analyses (e.g., calculating the mean mayfly length)				
Provided a clear explanation of their research methods, questions, and hypotheses				
Provided an adequate graph/data representation				

In these two studies on the feeding patterns of smallmouth bass and mayflies, we told you that “two groups of biologists” were carrying out the research. Who did you picture when you were thinking of the "biologists"?

Students
Expert scientists
Other (Please describe in box):

__________________________________________________

In this part of the survey, you will read information and answer questions about two studies depicting a predator‐prey relationship between great‐horned owls and house mice. Both groups are conducting independent investigations. You can go back to previous pages of the survey using the red “back” arrow on the bottom of the page, if needed. Two groups of biologists are studying the feeding behavior of the house mouse (Mus musculus, hereafter referred to as mouse/mice) in the presence or absence of one of its natural predators, the great‐horned owl (Bubo virginianus). Mice have a strong sense of smell and hearing and can be social or solitary, depending on living conditions. Mice commonly feed on seeds. Both species are nocturnal and generally feed at night. The two groups of biologists want to know how the presence of a great‐horned owl influences the amount of time that mice spend feeding.

### A.4 Group 1's study

We trapped 10 mice from multiple nearby fields. We brought them into the laboratory and set up two cages each containing five mice and a rodent nest box where mice can hide, burrow, and sleep. A bowl with a large amount of seeds was placed outside the nest. We placed infrared cameras in the cages to record mouse behavior over one night and watched the video to determine the time the mice spent at the food bowl.

One mouse cage was placed in Room 1 and one mouse cage was placed in Room 2. In Room 1, mouse behavior was recorded as they moved in and out of the nest box. In Room 2, we played 30‐s great‐horned owl calls every 15 min and recorded mouse behavior as they moved in and out of the nest box.

*Below are pictures of the laboratory setup in Rooms 1 and 2*.

We calculated the mean (average) amount of time the five mice in each room spent at their food bowl and found the following pattern:

*Note: Error bars indicate standard deviation*

What do you think Group 1 should say about the feeding behavior of mice while great‐horned owl calls play?

Mice spend less time at the food bowl in the presence of an owl predator call.
Mice spend more time at the food bowl in the presence of an owl predator call.
Mice spend the same amount of time as they usually do at the food bowl in the presence of an owl predator call.
There is not enough evidence to determine mouse feeding behavior.

Please explain your reasoning for your choice in the space below:

________________________________________________________________

How effective was Group 1, overall, in testing the feeding behavior of mice while great‐horned owl calls play?

Ineffective (1)
2
3
Effective (4)

What should Group 1 **do next**? (S*elect up to 3 options total.)*

Redesign the study to run for a longer period of time
Show a visual of an owl while owl calls play
Increase the number of mice in the study
Separate the mice into individual cages
Control for other biological variables (i.e., biotic factors)
Account for human error
Redesign the study for an outdoor setting
Sample mice from other fields
Conduct statistical analyses
Run a study where a variable is manipulated
Repeat the study to gather more data
Control for non‐biological variables (i.e., abiotic factors)
Other (Please describe in the box):

__________________________________________________

Below is information about the second group of biologists studying great‐horned owls and house mice. As a reminder, mice have a strong sense of smell and hearing and can be social or solitary, depending on living conditions. Mice commonly feed on seeds. Both species are nocturnal and generally feed at night. The two groups of biologists want to know how the presence of a great‐horned owl influences the amount of time that mice spend feeding.

### A.5 Group 2's study

We set up 15 cages in an outdoor enclosure. Each cage has one mouse and a rodent nest box where mice can hide, burrow, and sleep. The mice were trapped from a single nearby field. A bowl with a large amount of seeds was placed outside the nest. We conducted our study across two nights. We placed infrared cameras in the cages to record mouse behavior throughout these nights and watched the video to determine the time the mice spent at their food bowls.

On night one, mouse behavior was recorded in the absence of a predator.

On night two, we placed one great‐horned owl in the outdoor enclosure to measure mouse behavior in the presence of a predator. The owl was able to freely fly around the enclosure and could rest in trees, but it could not access the caged mice. The mice were able to view, smell, and hear the owl.

*Below is a picture of Group 2's fieldsite and an example of one of the cages*.

We determined the total amount of time each mouse spent at their food bowl per night and found the following pattern:

*Reminder: Each circle equals the total amount of time a mouse spent at the food bowl*.

What do you think Group 2 should say about the feeding behavior of the mice in the presence of a great‐horned owl?

Mice spend less time at the food bowl in the presence of an owl predator.
Mice spend more time at the food bowl in the presence of an owl predator.
Mice spend the same amount of time as they usually do at the food bowl in the presence of an owl predator.
There is not enough evidence to determine mouse feeding behavior.

Please explain your reasoning for your choice in the space below:

________________________________________________________________

How effective was Group 2, overall, in testing the feeding behavior of mice in the presence of a great‐horned owl?

Ineffective (1)
2
3
Effective (4)

What should Group 2 **do next**? (S*elect up to 3 options total.)*

Redesign the study to run for a longer period of time
Add a different type of predator alongside the owl
Increase the number of mice in the study
Control for other biological variables (i.e., biotic factors)
Account for human error
Redesign the study for a controlled laboratory environment
Sample mice from other fields
Add a different type of predator instead of the owl
Conduct statistical analyses
Run a study where a variable is manipulated
Repeat the study to gather more data
Control for non‐biological variables (i.e., abiotic factors)
Other (Please describe in the box): __________________________________________________

Which group do you think gained a more accurate understanding of the feeding behavior of mice in the presence of a great‐horned owl?

Group 1
Group 2
Both groups gained an accurate understanding
Neither group gained an accurate understanding

How do you think **Group 1** and **Group 2** performed in the following categories?

**Table:**

	Group 1 was more effective	Group 2 was more effective	Both Groups were effective	Neither group was effective
Used an appropriate study setting (**Group 1:** cages in laboratory; **Group 2:** cages in outdoor enclosure)				
Used an appropriate duration of time for the study (**Group 1:** one night; **Group 2:** two nights)				
Used an appropriate study setup (**Group 1:** Rooms with and without owl calls; **Group 2:** Nights with and without owl present)				
Placed an appropriate number of mice in each cage (**Group 1:** 5 mice per cage; **Group 2:** 1 mouse per cage)				
Used appropriate methods to collect data (e.g., measuring time at food bowl)				
Selected a sufficient sample size (**Group 1:** 10 total mice **Group 2:** 15 total mice)				

How do you think **Group 1** and **Group 2** performed in the following categories?

**Table:**

	Group 1 was more effective	Group 2 was more effective	Both Groups were effective	Neither group was effective
Used appropriate methods to record mouse behaviors (i.e., infrared cameras to record mouse behavior)				
Represented the predator appropriately (**Group 1:** owl calls; **Group 2:** live owl)				
Used appropriate sampling methods (**Group 1:** Collecting mice from multiple nearby fields; **Group 2:** Collecting mice from a single nearby field)				
Provided a clear explanation of their research methods, questions, and hypotheses				
Ran appropriate analyses (**Group 1:** average time at food bowl per cage; **Group 2:** total time at food bowl per cage each night)				
Provided an adequate graph/data representation				

In these two studies on the feeding behavior of mice in the presence or absence of owls, we told you that “two groups of biologists” were carrying out the research. Who did you picture when you were thinking of the "biologists"?

Students
Expert scientists
Other (Please describe in box):

__________________________________________________

### A.6 Demographic Questions

**Instructions:** Please answer the following questions to the best of your ability. Your answers will be used to better understand the characteristics of students taking this survey.

Please indicate how well you agree with the following statements:

**Table:**

	Strongly disagree	Somewhat disagree	Neither agree nor disagree	Somewhat agree	Strongly agree
**When I was younger**, I spent a lot of time in natural areas (e.g., exploring and hiking).					
**Now**, I spend a lot of time in natural areas (e.g., exploring and hiking).					
**I have prior experience** conducting field research (collecting data outdoors on natural phenomena).					
**I want more experience** conducting field research (collecting data outdoors on natural phenomena).					

Have you participated in undergraduate research as part of a faculty member's research group? If so, for how many terms (term = 1 semester or 1 quarter or 1 summer)?

1–2 terms
3–4 terms
5–6 terms
6+ terms
I have not conducted research in a faculty member's research group

If you have participated in undergraduate research in a faculty member's research group, where was most of your work completed?

Predominantly outdoors (i.e., field research)
Predominantly in a laboratory environment
Both in the field and the laboratory

How confident are you in your ability to read and interpret scientific graphs?

Not confident (1)
2
3
4
Confident (5)

Where would you put doing **biology FIELD studies** on the following scales between two opposite adjectives (5‐point scale)?

**Table:**

	1	2	3	4	5	
	1	2	3	4	5	
Boring						Interesting
Useless						Useful
Hard						Easy
Dangerous						Safe

Are you 18 years of age or older?

Yes
No
Prefer not to disclose

What is your current class standing?

First year
Sophomore
Junior
Senior
Postbaccalaureate
Graduate student
Other ________________________________________________
Prefer not to disclose

Which gender do you identify most closely with? (*Select all that apply*)

Man
Woman
Non‐binary / Non‐gender conforming
Self‐describe (describe in the box): __________________________________________________
Prefer not to disclose

What is your race/ethnicity? (*Select all that apply*)

American Indian or Alaska Native
Asian
Black or African American
Hispanic or Latinx
Native Hawaiian / Pacific Islander
White
Self‐describe (describe in the box): __________________________________________________
Prefer not to disclose

How many college‐level biology courses have you taken that include a fieldwork/outdoor experiment component, including any in which you are currently enrolled?

0
1–2
3–4
5+
Prefer not to disclose

Have you declared or are you planning to declare a major in biology or another life science?

Yes
No
Prefer not to disclose

Which of the subdisciplines of biology do you intend to focus on?

Ecology & Evolutionary Biology
Molecular Biology (e.g., Cell biology, Biochemistry, Developmental Biology)
Physiology/Neuroscience
No specialization / Don't know

What is your primary or intended major/field?

Non‐biology science (e.g., physical sciences, chemistry)
Technology (e.g., information science, computer science)
Engineering
Mathematics
Pre‐health profession (e.g., pre‐med, pre‐pharmacy, pre‐vet)
Humanities
Social sciences
Other (describe in the box): __________________________________________________

Highest level of education completed by at least one of your parent(s):

Did not complete high school
High school/GED
Some college (but did not complete college)
Associate's degree (2‐year degree)
Bachelor's degree
Master's degree
Advanced graduate degree (for example, DVM, MD, Ph.D.)
Not sure
Prefer not to disclose

Please indicate how well you agree with the following statements:

**Table:**

	Strongly disagree	Somewhat disagree	Neither agree nor disagree	Somewhat agree	Strongly agree
Doing well on this survey was important to me.					
The questions on this survey were easy.					
I engaged in good effort throughout this survey.					
I tried hard on the questions at the start and then got bored.					
I am curious about how I did on this survey compared with others.					
While answering the questions, I could have worked harder on them.					
I did not give this survey my full attention while completing it.					
I would like to know how well I did on this survey.					
I feel confident in my answers to this survey.					
I just clicked through the questions and chose randomly to get the participation credit.					
I am not concerned about the score I receive on this survey.					
I tried my best on all or most of the questions.					

What is your approximate current overall/cumulative G.P.A.?

0.0–0.69 (E or F)
display 0.7–1.69 (D− to D+)
1.7–2.69 (C− to C+)
2.7–3.69 (B− to B+)
3.7–4.00+ (A− to A+)
Prefer not to disclose

In order to receive credit, please provide your first name, last name, and your school ID number. This information is used to record your participation.
