# Supplementary material for: Comparing study features is easy but identifying next steps is hard: Evaluating critical thinking through the Biology Lab Inventory of Critical Thinking in Ecology
Source: Ecol Evol. 2023 May 10;13(5):e10071. doi: 10.1002/ece3.10071 (PMC10171991; doi:10.1002/ece3.10071)
Supplement: Supplementary file 1 — Appendix [file ECE3-13-e10071-s001.docx]

Appendix 1. Eco-BLIC.

The following questions will ask you about case studies where groups are exploring ecological environments. The aggregate results will be used to help develop the content for your biology course.

Please do NOT use outside resources to answer the questions.  

[Insert consent form here]

**In this part of the survey, you will read information and answer questions about two studies depicting a predator-prey relationship between smallmouth bass and mayflies. Both groups are conducting independent investigations.** **You can go back to previous pages of the survey using the red “back” arrow on the bottom of the page, if needed.** Two groups of biologists are studying smallmouth bass (Micropterus dolomieu) and combmouthed minnow mayflies (Ameletus cryptostimulus; hereafter referred to as mayflies). Smallmouth bass eat young mayflies, which live at the water surface. Mayflies do not grow bigger than what smallmouth bass can eat. Both groups of biologists want to know whether smallmouth bass selectively feed on larger or smaller mayflies.

Group 1's Study
We conducted surveys of nearby 30 ponds: 15 ponds that contained smallmouth bass and 15 ponds that contained no smallmouth bass.

*Below you will find a picture of one of Group 1’s study sites*


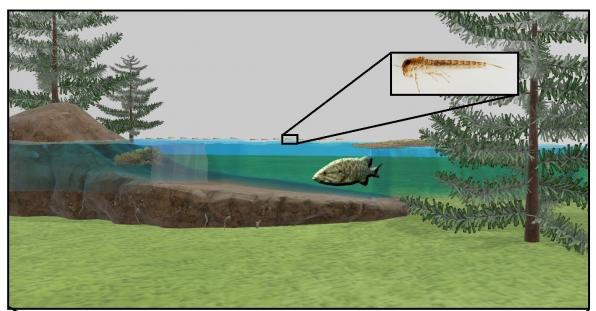
We measured 10 young mayflies within each pond and calculated the mean length of mayflies for each. We found the following pattern:​
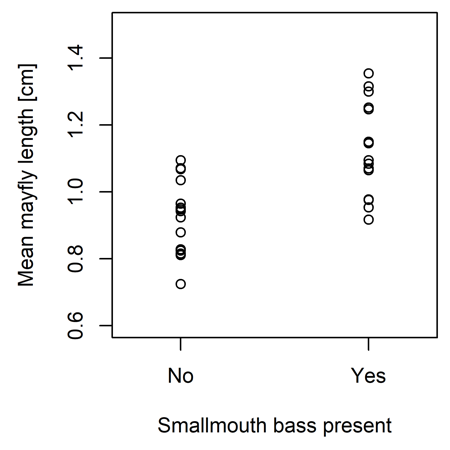

                                          
*Reminder: Each circle equals the mean (average) mayfly length per pond.*

What do you think Group 1 should say about the feeding pattern between smallmouth bass and mayflies?

- Smallmouth bass selectively consume smaller mayflies.
- Smallmouth bass selectively consume larger mayflies.
- Smallmouth bass consume mayflies with no size-preference.
- There is not enough evidence to determine the feeding pattern.

Please explain your reasoning in the space below.

________________________________________________________________

How effective was Group 1, overall, in testing whether smallmouth bass selectively feed on larger or smaller mayflies?

- Ineffective (1)
- 2
- 3
- Effective (4)

What should Group 1 **do next**? (S*elect up to 3 options total.)*

- Redesign the study to run for a longer period of time
- Repeat the study using organisms from a randomly selected set of ponds
- Control for biological variables (i.e., biotic factors)
- Account for human error
- Redesign the study for a controlled laboratory environment
- Sample from more ponds
- Conduct statistical analyses
- Run a study where a variable is manipulated
- Repeat the study to gather more data
- Control for non-biological variables (i.e., abiotic factors)
- Other (Please describe in the box): __________________________________________________

**Below is information about the second group of biologists studying smallmouth bass and mayflies. As a reminder, smallmouth bass eat young mayflies, which live at the water surface. Mayflies do not grow bigger than what smallmouth bass can eat. Both groups of biologists want to know whether smallmouth bass selectively feed on larger or smaller mayflies.**

Group 2's study
We collected smallmouth bass and young mayflies from a single pond. We established ten tanks and placed 100 mayflies in each tank. We placed one smallmouth bass in each tank for 24 hours. The tanks are covered with a net, so the mayflies cannot move to different tanks.

*Below is a picture of Group 2’s laboratory setup.*


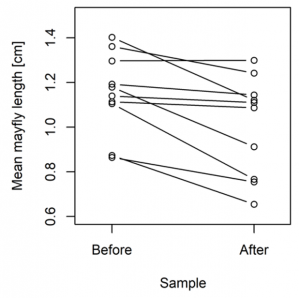

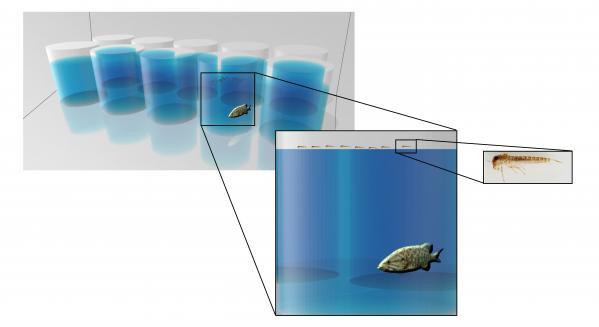


We took a random sample of 20 mayflies from each tank before and after the 24-hour period. We calculated the mean (average) length of mayflies in each sample. We found the following pattern:​
                                        
                                             
*Reminder: Each circle equals the mean (average) mayfly length per tank. Matched samples are connected with a solid line.*

What do you think Group 2 should say about the feeding pattern between smallmouth bass and mayflies?

- Smallmouth bass selectively consume smaller mayflies.
- Smallmouth bass selectively consume larger mayflies.
- Smallmouth bass consume mayflies with no size-preference.
- There is not enough evidence to determine the feeding pattern.

Please explain your reasoning in the space below.

________________________________________________________________

How effective was Group 2, overall, in testing whether smallmouth bass selectively feed on larger or smaller mayflies?

- Ineffective (1)
- 2
- 3
- Effective (4)

What should Group 2 **do next**? (S*elect up to 3 options total.)*

- Redesign the study to run for a longer period of time
- Increase the number of tanks used in the laboratory
- Control for other biological variables (i.e, biotic factors)
- Account for human error
- Redesign the study for an outdoor setting
- Sample from more ponds
- Conduct statistical analyses
- Run a study where a variable is manipulated
- Repeat the study to gather more data
- Control for non-biological variables (i.e., abiotic factors)
- Other (Please describe in the box): __________________________________________________

Which group do you think gained a more accurate understanding of the feeding pattern between smallmouth bass and mayflies?

- Group 1
- Group 2
- Both groups gained an accurate understanding
- Neither group gained an accurate understanding

How do you think **Group 1** and **Group 2** performed in the following categories?

|  | Group 1 was more effective | Group 2 was more effective | Both Groups were effective | Neither group was effective |
| --- | --- | --- | --- | --- |
| Used an appropriate study setting (**Group 1:** ponds; **Group 2:** tanks) |  |  |  |  |
| Used an appropriate study setup (**Group 1:** Ponds with and without bass; **Group 2:** Tanks before and after bass) |  |  |  |  |
| Used appropriate methods to collect data (e.g., measuring mayfly length) |  |  |  |  |
| Selected a sufficient sample size (**Group 1:** 10 mayflies per pond; **Group 2:** 20 mayflies per tank) |  |  |  |  |

How do you think **Group 1** and **Group 2** performed in the following categories?

|  | Group 1 was more effective | Group 2 was more effective | Both Groups were effective | Neither group was effective |
| --- | --- | --- | --- | --- |
| Sampled from an appropriate number of ponds (**Group 1:** 30 ponds; **Group 2:** 1 pond) |  |  |  |  |
| Ran appropriate analyses (e.g., calculating the mean mayfly length) |  |  |  |  |
| Provided a clear explanation of their research methods, questions, and hypotheses |  |  |  |  |
| Provided an adequate graph / data representation |  |  |  |  |

In these two studies on the feeding patterns of smallmouth bass and mayflies, we told you that 'two groups of biologists' were carrying out the research. Who did you picture when you were thinking of the "biologists"?

- Students
- Expert scientists
- Other (Please describe in box): __________________________________________________

**In this part of the survey, you will read information and answer questions about two studies depicting a predator-prey relationship between great-horned owls and house mice. Both groups are conducting independent investigations. You can go back to previous pages of the survey using the red “back” arrow on the bottom of the page, if needed. Two groups of biologists are studying the feeding behavior of the house mouse (Mus musculus, hereafter referred to as mouse/mice) in the presence or absence of one of its natural predators, the great-horned owl (Bubo virginianus). Mice have a strong sense of smell and hearing and can be social or solitary, depending on living conditions. Mice commonly feed on seeds. Both species are nocturnal and generally feed at night. The two groups of biologists want to know how the presence of a great-horned owl influences the amount of time that mice spend feeding.**


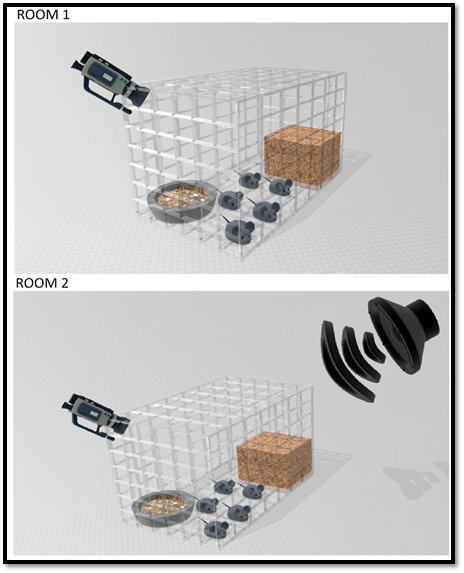
Group 1's study
We trapped 10 mice from multiple nearby fields. We brought them into the lab and set up two cages each containing five mice and a rodent nest box where mice can hide, burrow, and sleep. A bowl with a large amount of seeds was placed outside the nest. We placed infrared cameras in the cages to record mouse behavior over one night and watched the video to determine the time the mice spent at the food bowl. 

One mouse cage was placed in Room 1 and one mouse cage was placed in Room 2. In Room 1, mouse behavior was recorded as they moved in and out of the nest box. In Room 2, we played 30-second great-horned owl calls every 15 minutes and recorded mouse behavior as they moved in and out of the nest box.

*Below are pictures of the laboratory setup in Rooms 1 and 2.*
**We calculated the mean (average) amount of time the five mice in each room spent at their food bowl and found the following pattern:**

                                          
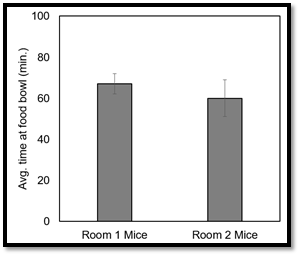

*Note: Error bars indicate standard deviation*

What do you think Group 1 should say about the feeding behavior of mice while great-horned owl calls play?

- Mice spend less time at the food bowl in the presence of an owl predator call.
- Mice spend more time at the food bowl in the presence of an owl predator call.
- Mice spend the same amount of time as they usually do at the food bowl in the presence of an owl predator call.
- There is not enough evidence to determine mouse feeding behavior.

Please explain your reasoning for your choice in the space below:

________________________________________________________________

How effective was Group 1, overall, in testing the feeding behavior of mice while great-horned owl calls play?

- Ineffective (1)
- 2
- 3
- Effective (4)

What should Group 1 **do next**? (S*elect up to 3 options total.)*

- Redesign the study to run for a longer period of time
- Show a visual of an owl while owl calls play
- Increase the number of mice in the study
- Separate the mice into individual cages
- Control for other biological variables (i.e., biotic factors)
- Account for human error
- Redesign the study for an outdoor setting
- Sample mice from other fields
- Conduct statistical analyses
- Run a study where a variable is manipulated
- Repeat the study to gather more data
- Control for non-biological variables (i.e., abiotic factors)
- Other (Please describe in the box): __________________________________________________

**Below is information about the second group of biologists studying great-horned owls and house mice. As a reminder, mice have a strong sense of smell and hearing and can be social or solitary, depending on living conditions. Mice commonly feed on seeds. Both species are nocturnal and generally feed at night. The two groups of biologists want to know how the presence of a great-horned owl influences the amount of time that mice spend feeding.**


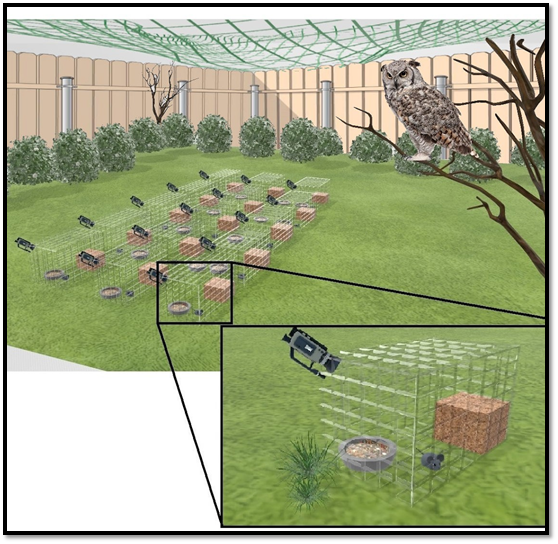
Group 2's study
We set up 15 cages in an outdoor enclosure. Each cage has one mouse and a rodent nest box where mice can hide, burrow, and sleep. The mice were trapped from a single nearby field. A bowl with a large amount of seeds was placed outside the nest. We conducted our study across two nights. We placed infrared cameras in the cages to record mouse behavior throughout these nights and watched the video to determine the time the mice spent at their food bowls.

On night one, mouse behavior was recorded in the absence of a predator. 

On night two, we placed one great-horned owl in the outdoor enclosure to measure mouse behavior in the presence of a predator. The owl was able to freely fly around the enclosure and could rest in trees, but it could not access the caged mice. The mice were able to view, smell, and hear the owl.
          
*Below is a picture of Group 2’s fieldsite and an example of one of the cages.*


**We determined the total amount of time each mouse spent at their food bowl per night and found the following pattern:**
                  
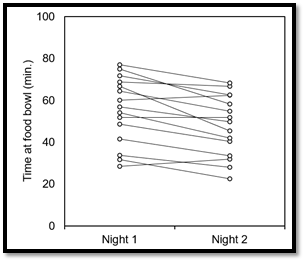
                            
*Reminder: Each circle equals the total amount of time a mouse spent at the food bowl*

What do you think Group 2 should say about the feeding behavior of the mice in the presence of a great-horned owl?

- Mice spend less time at the food bowl in the presence of an owl predator.
- Mice spend more time at the food bowl in the presence of an owl predator.
- Mice spend the same amount of time as they usually do at the food bowl in the presence of an owl predator.
- There is not enough evidence to determine mouse feeding behavior.

Please explain your reasoning for your choice in the space below:

________________________________________________________________

How effective was Group 2, overall, in testing the feeding behavior of mice in the presence of a great-horned owl?

- Ineffective (1)
- 2
- 3
- Effective (4)

What should Group 2 **do next**? (S*elect up to 3 options total.)*

- Redesign the study to run for a longer period of time
- Add a different type of predator alongside the owl
- Increase the number of mice in the study
- Control for other biological variables (i.e, biotic factors)
- Account for human error
- Redesign the study for a controlled laboratory environment
- Sample mice from other fields
- Add a different type of predator instead of the owl
- Conduct statistical analyses
- Run a study where a variable is manipulated
- Repeat the study to gather more data
- Control for non-biological variables (i.e., abiotic factors)
- Other (Please describe in the box): __________________________________________________

Which group do you think gained a more accurate understanding of the feeding behavior of mice in the presence of a great-horned owl?

- Group 1
- Group 2
- Both groups gained an accurate understanding
- Neither group gained an accurate understanding

How do you think **Group 1** and **Group 2** performed in the following categories?

|  | Group 1 was more effective | Group 2 was more effective | Both Groups were effective | Neither group was effective |
| --- | --- | --- | --- | --- |
| Used an appropriate study setting (**Group 1:** cages in lab; **Group 2:** cages in outdoor enclosure) |  |  |  |  |
| Used an appropriate duration of time for the study (**Group 1:** one night; **Group 2:** two nights) |  |  |  |  |
| Used an appropriate study setup (**Group 1:** Rooms with and without owl calls; **Group 2:** Nights with and without owl present) |  |  |  |  |
| Placed an appropriate number of mice in each cage (**Group 1:**5 mice per cage; **Group 2:**1 mouse per cage) |  |  |  |  |
| Used appropriate methods to collect data (e.g., measuring time at food bowl) |  |  |  |  |
| Selected a sufficient sample size (**Group 1:** 10 total mice **Group 2:** 15 total mice) |  |  |  |  |

How do you think **Group 1** and **Group 2** performed in the following categories?

|  | Group 1 was more effective | Group 2 was more effective | Both Groups were effective | Neither group was effective |
| --- | --- | --- | --- | --- |
| Used appropriate methods to record mouse behaviors (i.e., infrared cameras to record mouse behavior) |  |  |  |  |
| Represented the predator appropriately (**Group 1:** owl calls; **Group 2:** live owl) |  |  |  |  |
| Used appropriate sampling methods (**Group 1:**Collecting mice from multiple nearby fields; **Group 2:**Collecting mice from a single nearby field) |  |  |  |  |
| Provided a clear explanation of their research methods, questions, and hypotheses |  |  |  |  |
| Ran appropriate analyses (**Group 1:** average time at food bowl per cage; **Group 2:** total time at food bowl per cage each night) |  |  |  |  |
| Provided an adequate graph / data representation |  |  |  |  |

In these two studies on the feeding behavior of mice in the presence or absence of owls, we told you that 'two groups of biologists' were carrying out the research. Who did you picture when you were thinking of the "biologists"?

- Students
- Expert scientists
- Other (Please describe in box): __________________________________________________

**Demographic Questions**

**Instructions:** Please answer the following questions to the best of your ability. Your answers will be used to better understand the characteristics of students taking this survey.

Please indicate how well you agree with the following statements:

|  | Strongly disagree | Somewhat disagree | Neither agree nor disagree | Somewhat agree | Strongly agree |
| --- | --- | --- | --- | --- | --- |
| **When I was younger**, I spent a lot of time in natural areas (e.g., exploring, hiking). |  |  |  |  |  |
| **Now**, I spend a lot of time in natural areas (e.g., exploring, hiking). |  |  |  |  |  |
| **I have prior experience** conducting field research (collecting data outdoors on natural phenomena). |  |  |  |  |  |
| **I want more experience** conducting field research (collecting data outdoors on natural phenomena). |  |  |  |  |  |

Have you participated in undergraduate research as part of a faculty member’s research group? If so, for how many terms (term = 1 semester or 1 quarter or 1 summer)?

- 1-2 terms
- 3-4 terms
- 5-6 terms
- 6+ terms
- I have not conducted research in a faculty member's research group

If you have participated in undergraduate research in a faculty member’s research group, where was most of your work completed?

- Predominantly outdoors (i.e., field research)
- Predominantly in a laboratory environment
- Both in the field and the lab

How confident are you in your ability to read and interpret scientific graphs?

- Not confident (1)
- 2
- 3
- 4
- Confident (5)

Where would you put doing **biology FIELD studies** on the following scales between two opposite adjectives (5 point scale)?

|  | 1 | 2 | 3 | 4 | 5 |  |
| --- | --- | --- | --- | --- | --- | --- |
|  | 1 | 2 | 3 | 4 | 5 |  |
| Boring |  |  |  |  |  | Interesting |
| Useless |  |  |  |  |  | Useful |
| Hard |  |  |  |  |  | Easy |
| Dangerous |  |  |  |  |  | Safe |

Are you 18 years of age or older?

- Yes
- No
- Prefer not to disclose

What is your current class standing?

- First year
- Sophomore
- Junior
- Senior
- Postbaccalaureate
- Graduate student
- Other __________________________________________________
- Prefer not to disclose

Which gender do you identify most closely with? (*Select all that apply*)

- Man
- Woman
- Non-binary / Non-gender conforming
- Self-describe (describe in the box): __________________________________________________
- Prefer not to disclose

What is your race/ethnicity? (*Select all that apply*)

- American Indian or Alaska Native
- Asian
- Black or African American
- Hispanic or Latinx
- Native Hawaiian / Pacific Islander
- White
- Self-describe (describe in the box): __________________________________________________
- Prefer not to disclose

How many college-level biology courses have you taken that include a fieldwork/outdoor experiment component, including any in which you are currently enrolled?

- 0
- 1-2
- 3-4
- 5+
- Prefer not to disclose

Have you declared or are you planning to declare a major in biology or another life science?

- Yes
- No
- Prefer not to disclose

Which of the subdisciplines of biology do you intend to focus on?

- Ecology & Evolutionary Biology
- Molecular Biology (e.g., Cell biology, Biochemistry, Developmental Biology)
- Physiology/Neuroscience
- No specialization / Don't know

What is your primary or intended major/field?

- Non-biology science (e.g., physical sciences, chemistry)
- Technology (e.g., information science, computer science)
- Engineering
- Mathematics
- Pre-health profession (e.g., pre-med, pre-pharmacy, pre-vet)
- Humanities
- Social sciences
- Other (describe in the box): __________________________________________________

Highest level of education completed by at least one of your parent(s):

- Did not complete high school
- High school/GED
- Some college (but did not complete college)
- Associate's degree (2 year degree)
- Bachelor's degree
- Master's degree
- Advanced graduate degree (for example, DVM, MD, Ph.D.)
- Not sure
- Prefer not to disclose

Please indicate how well you agree with the following statements:

|  | Strongly disagree | Somewhat disagree | Neither agree nor disagree | Somewhat agree | Strongly agree |
| --- | --- | --- | --- | --- | --- |
| Doing well on this survey was important to me. |  |  |  |  |  |
| The questions on this survey were easy. |  |  |  |  |  |
| I engaged in good effort throughout this survey. |  |  |  |  |  |
| I tried hard on the questions at the start and then got bored. |  |  |  |  |  |
| I am curious about how I did on this survey compared to others. |  |  |  |  |  |
| While answering the questions, I could have worked harder on them. |  |  |  |  |  |
| I did not give this survey my full attention while completing it. |  |  |  |  |  |
| I would like to know how well I did on this survey. |  |  |  |  |  |
| I feel confident in my answers to this survey. |  |  |  |  |  |
| I just clicked through the questions and chose randomly to get the participation credit. |  |  |  |  |  |
| I am not concerned about the score I receive on this survey. |  |  |  |  |  |
| I tried my best on all or most of the questions. |  |  |  |  |  |

What is your approximate current overall/cumulative G.P.A.?

- 0.0 - 0.69 (E or F)
- 0.7 - 1.69 (D- to D+)
- 1.7 - 2.69 (C- to C+)
- 2.7 - 3.69 (B- to B+)
- 3.7 - 4.00+ (A- to A+)
- Prefer not to disclose

**In order to receive credit, please provide your first name, last name, and your school ID number. This information is used to record your participation.**
